# The relationship between nurses’ perceptions of spiritual care and their attitudes towards death and care of dying patients

**DOI:** 10.1017/S1478951526101813

**Published:** 2026-03-04

**Authors:** Sevde Yuksel, Zeliha Koç

**Affiliations:** 1 Boyabat State Hospital, Palliative Care Unit, Sinop, Türkiye; 2 Health Science Faculty, Ondokuz Mayıs University, Samsun, Türkiye

**Keywords:** nurse, spiritual care, death, attitude, spiritualite

## Abstract

**Background:**

Holistic nursing care requires considering not only the physical and psychosocial but also the spiritual care needs of the patient.

**Objectives:**

This study aimed to determine the relationship between nurses’ perceptions of the concept of spiritual care and their attitudes towards death and the care of dying patients.

**Methods:**

The sample of this descriptive and correlational study consisted of 383 nurses. Data were collected using the Spirituality and Spiritual Care Rating Scale (SSCRS), the Spiritual Support Perception Scale (SSPS), the Frommelt Attitude Toward Care of the Dying Scale (FATCOD), and the Death Attitude Profile Scale (DAP-R). The predictive role of nurses’ perceptions of spiritual care on their attitudes toward caregiving for patients approaching death and dying was examined using path analysis. The results of the analysis were presented as descriptive statistics (mean ± SD), and frequency (percentage) distributions. The reliability of the scales and subscales was examined using the Cronbach’s alpha internal consistency coefficient.

**Results:**

The path coefficient between the SSCRS and the DAP-R subscales of *Fear of Death* (β = 0.232; *P* = 0.025), *Death Avoidance* (β = 0.301; *P* = 0.007), *Neutral Acceptance* (β = 0.22; *P* = 0.01), *Approach Acceptance* (β = 0.444; *P* < 0.001), and *Escape Acceptance* (β = 0.659; *P* < 0.001) was statistically significant. The path coefficient between the FATCOD and the DAP-R subscales of *Fear of Death* (β = −0.032; *P* < 0.001), *Death Avoidance* (β = −0.038; *P* < 0.001), and *Neutral Acceptance* (β = 0.02; *P* < 0.001) was statistically significant.

**Significance of Results:**

It was determined that there was a correlation between the nurses’ perceptions of the concept of spiritual care and their attitudes towards death and the care of dying patients. It can be suggested that the nurses exhibited a more positive attitude toward the care of dying patients as their understanding of spirituality, spiritual support, and spiritual care increased.

## Introduction

Patients’ need for holistic care may increase when they are dying, which is the final stage of life (Potter et al. 2017). Holistic nursing care requires considering not only the physical and psychosocial but also the spiritual care needs of the patient (Wilkinson et al. 2016). Nurses generally recognize that patients have a spiritual dimension; however, they may avoid or ignore identifying and intervening in their spiritual problems (Taylor et al. 2015; Wilkinson et al. 2016; Byoung Sook and Su Young 2017; Potter et al. 2017; Kozier et al. 2018). They are usually able to focus on the physical aspect of care (Kisvetrova et al. 2018; Wisesrith et al. 2021).

## Background

Spiritual and existential care is an important component of holistic care (Tornoe et al. 2015; Bone et al. 2018; Kudubes et al. 2021). Individuals may turn to prayer and other spiritual practices when they are ill (Kozier et al. 2018). When patients experience an acute, chronic, or terminal illness, the energy they derive from their sense of spirituality can help them feel good about themselves, increasing their desire and strength to recover (Giske and Cone 2015; Potter et al. 2017). Nurses should thus improve their knowledge and awareness of the spiritual and religious beliefs and practices of their patients to meet their needs in this area (Taylor et al. 2015; Wilkinson et al. 2016; Potter et al. 2017; Kozier et al. 2018; Wisesrith et al. 2021).

Regardless of specific religious beliefs, anyone can have a sense of spirituality. This kind of spirituality can empower an individual to love, have faith and hope, search for meaning in life, and improve relationships with others (Potter et al. 2017). Paying attention to a patient’s spirituality helps to focus nursing practice holistically (Potter et al. 2017). To provide holistic nursing care, nurses must be sensitive to the spiritual needs of patients and meet these needs appropriately (Wilkinson et al. 2016; Kozier et al. 2018).

During clinical practice, nurses should maintain a positive attitude when providing care to dying patients and should have the ability to cope with the loss of a patient. Nurses who are not able to cope with patient death may not be competent in supporting dying patients and their family members, and the quality of care provided may decrease (Zheng et al. 2018). The process of coping with patient death is a personal experience and may differ for each nurse. It may be affected by nurses’ beliefs regarding, and attitudes towards, death, their fear of death, religious factors, previous training about death and dying, cultural characteristics, and their experience of providing care to dying patients (Wang et al. 2018; Zheng et al. 2018). Training programs can be implemented on topics such as death and dying, grief care, and grief counseling in order to increase the competence of nurses to cope with patient death (Zheng et al. 2018).

Spiritual care is an important and non-negligible dimension of nursing practice (Taylor et al. 2015; Wilkinson et al. 2016; Potter et al. 2017; Kozier et al. 2018). When nurses are aware of patients’ religious and spiritual beliefs and practices, they can better help them to meet their spiritual needs. Adopting a compassionate approach, nurses can support dying patients to find meaning and purpose despite their suffering, illness, and approaching death (Taylor et al. 2015; Wisesrith et al. 2021). Given that patients have the right to receive care that respects their spiritual and religious values, nurses should provide spiritual care consistent with patients’ beliefs (Kozier et al. 2018).

In health care practices, a respectful environment should be established that complies with human rights, values, traditions, and spiritual beliefs, and the dying individual and their family should be supported when and if they wish the traditional rituals surrounding death to be carried out (Taylor et al. 2015; Wilkinson et al. 2016; Potter et al. 2017; Kozier et al. 2018). In studies on this topic, it has been suggested that when nurses’ attitudes towards death are more negative, this reduces their level of perception of the concept of spiritual care (Kudubes et al. 2021), that nurses generally lack spiritual care competencies, and that nurses should gain these competencies and develop positive attitudes toward death to meet the spiritual needs of patients and improve their quality of life (Byoung Sook and Su Young 2017; Batstone et al. 2020; Li et al. 2021).

Health care team members need to evaluate the changing face of spiritual care and their own roles in providing such care to patients (Sulmasy 2012). It is very important that nurses develop an understanding of spiritual care based on the general characteristics of spirituality (Kang et al. 2021). A limited number of studies has been conducted in Türkiye to determine nurses’ perceptions of the concept of spiritual care or their attitudes towards death (Çevik and Kav 2013; Gurdogan et al. 2017; Karadag et al. 2019; Pehlivan et al. 2020; Kudubes et al. 2021; Rahman et al. 2021; İnci and Sözen 2024). Research findings may be useful in organizing training to increase the spiritual care competencies of nurses and in developing clinical practice guidelines regarding spiritual care (Kang et al. 2021). These activities may help nurses improve their attitudes towards spiritual care and increase their self-awareness (Adip-Hajbaghery et al. 2017). They may also guide nurse educators and researchers in evaluating nursing curricula. This study aimed to determine the relationship between nurses’ perceptions of the concept of spiritual care and their attitudes towards death and the care of dying patients.

1. **Hypothesis**

**H_0_**: There is no relationship between nurses’ perceptions of spiritual care and their attitudes towards caregiving for patients approaching death and dying.

**H_1_:** There is a relationship between nurses’ perceptions of spiritual care and their attitudes towards caregiving for patients approaching death and dying.

### Theoretical framework

One of the most important goals of palliative care services is to reduce the psychological effects of the disease on the patient and their relatives, to enable the patient to cope with emotional difficulties, and to provide medical, psychological, social, and spiritual support. Interventions in palliative care include physical, psychosocial, and spiritual aspects. Because of this, spiritual care is an important component of palliative care service delivery (Chen et al. 2016). Accordingly, the conceptual framework of the study is based on Watson’s “Theory of Human Caring.” This theory emphasizes the importance of the philosophy of nursing care, especially focusing on spiritual and psychosocial dimensions. It focuses on the interpersonal process between the individual and the nurse, taking a holistic approach to the care of the individual (Masters 2015). Watson identified 10 healing caritas processes in the Theory of Human Caring. The 8th caritas process emphasizes the creation of a supportive, protective, and/or regulating mental, physical, sociocultural, and spiritual environment. The 10th caritas process focuses on allowing existential–phenomenological–spiritual forces (participating in the spiritual/mysterious and unknown existential dimensions of the individual’s life and death pain; providing spiritual care) (Watson 2010).

## Methods

### Study design and sampling method

This descriptive and correlational study was carried out in a university hospital between July 28, 2021, and March 30, 2023. G*Power version 3.1.9.6 was used to determine the number of nurses to be included in the study. Since the effect size, sample size, and α significance level were known, G*power post hoc statistical power analysis was used. The standardized effect size was calculated with reference to a previous study on this subject (Rahman et al. 2021). The minimum number of cases to be included in the study was determined as 383, with 95% confidence (1−α), 94% test power (1−β), and an effect size of f = 0.160. The DAP-R Fear of Death, Death Avoidance, Neutral Acceptance, Approach Acceptance, and Escape Acceptance sub-dimension mean scores were the dependent variables. The SSCRS, FATCOD, SSPS mean scores were the independent variables. Female and male nurses who had been employed as a nurse for at least one year, were not on sick leave or on leave on the dates the data were collected, were willing to participate in the study, and who completed the descriptive information form and scales were included in the study.

### Data collection

The principles of the Declaration of Helsinki were adhered to during data collection. The nurses were informed about the nature and purpose of the study. Verbal informed consent was obtained from the nurses. On appropriate days and at appropriate times, 400 data collection forms were distributed face-to-face to those nurses who were willing to participate. The nurses were asked to fill in the data collection forms completely. Seventeen forms distributed to the nurses were canceled due to missing data. The data collection process was terminated with the participation of 383 nurses.

### Data collection tools

#### Nurse descriptive information form

The Nurse Descriptive Information Form consisted of questions regarding the sociodemographic and professional characteristics of the nurses (age, marital status, gender, educational status, duration of employment, unit of employment, employment type, status of liking the profession, manner of working, mean number of patients cared for per day).

#### Spirituality and Spiritual Care Rating Scale

The Spirituality and Spiritual Care Rating Scale (SSCRS) was developed by McSherry et al. to identify individuals’ perceptions of the concepts of spirituality and spiritual care (McSherry et al. 2002). The Turkish validity and reliability study of the scale was conducted by Ergül and Bayık Temel (Ergül and Bayık Temel 2007). The SSCRS is a Likert-type scale consisting of 17 items. Thirteen items are scored directly, and 4 items are reverse-scored (items 3, 4, 13, and 16). The total score is calculated by dividing the score from all the items by the number of questions. A total mean score approaching 5 indicates an increase in the level of perception of the concepts of spirituality and spiritual care. The Cronbach’s alpha reliability coefficient of the scale was determined to be 0.76 (Ergül and Bayık Temel 2007). In this study, the Cronbach’s alpha reliability coefficient of the scale was calculated as 0.786.

#### Spiritual Support Perception Scale

The Spiritual Support Perception Scale (SSPS) was developed by Erkan Kavas and Nurgül Kavas (2014) to measure health professionals’ perceptions of the concept of spiritual support (Kavas and Kavas 2014). The SSPS is a Likert-type scale consisting of 15 items. The total scale score is determined by summing the scores from the items. The highest score obtainable from the scale is 60. An increase in the mean total score indicates an increase in the awareness of the concepts of spiritual support and spiritual care. A score between 0 and 20 points indicates a low level of awareness; a score between 20 and 40 points indicates a moderate level of awareness; a score between 40 and 60 points indicates a high level of awareness. The Cronbach’s alpha reliability coefficient of the scale was determined to be 0.940 (Kavas and Kavas 2014). In this study, the Cronbach alpha reliability coefficient of SSPS was calculated as 0.940.

#### Frommelt Attitude Toward Care of the Dying Scale

The Frommelt Attitude Toward Care of the Dying Scale (FATCOD) was developed by Katherine H. Murray Frommelt to explore how non-family caregivers involved in the care of dying patients feel about the care (Frommelt 2003). The Turkish validity and reliability study of the scale was conducted by Çevik and Kav (Çevik and Kav 2013). The FATCOD is a 5-point Likert-type scale consisting of 30 items. In evaluating the scale, questions about negative attitudes are reversed and their scores are summed with the scores from positive answers to obtain a total score. The total score obtainable from the scale varies between 30 and 150. A higher score indicates a more positive attitude toward the care of dying patients. The Cronbach’s alpha reliability coefficient of the FATCOD was determined to be 0.73 (Çevik and Kav 2013). In this study, the Cronbach’s alpha reliability coefficient of the FATCOD was calculated as 0.656.

#### Death Attitude Profile-Revised

The Death Attitude Profile-Revised (DAP-R) was developed by Wong et al. to evaluate participants’ attitudes toward death (Wong et al. 1994). The Turkish validity and reliability study of the scale was conducted by Çevik and Kav (2013). The DAP-R is a 7-point Likert-type scale consisting of 32 items. The scale consists of 5 subscales: Fear of Death, Death Avoidance, Neutral Acceptance, Approach Acceptance, and Escape Acceptance. *Fear of Death* refers to negative thoughts and feelings about death (items 1, 2, 7, 18, 20, 21, and 32). *Death Avoidance* refers to the participant’s attempts to avoid thoughts of death as much as possible (items 3, 10, 12, 19, and 26). *Neutral Acceptance* implies that the participant neither welcomes nor fears death (items 6, 14, 17, 24, and 30). *Approach Acceptance* indicates that the participant perceives death as a happy gateway to the afterlife (items 4, 8, 13, 15, 16, 22, 25, 27, 28, and 31). *Escape Acceptance* implies that the participant perceives death as an escape from a painful existence (items 5, 9, 11, 23, and 29). Scores for the 5 subscales are calculated by summing the score for each item and dividing the result by the number of items in that subscale. A high scale score indicates that the individual has a negative attitude toward death. The Cronbach’s alpha reliability coefficient of the scale was determined to be 0.80 (Çevik and Kav 2013). In this study, the Cronbach’s alpha reliability coefficient of DAP-R was calculated as 0.910.

### Ethical considerations

The study was initiated after receiving approval from the Clinical Research Ethics Committee of Ondokuz Mayıs University (B.30.2.ODM.0.20.08/407-902). For data collection, written permission was obtained from the Directorate of Ondokuz Mayıs University Health Practice and Research Center, where the study was conducted (E-15374210-302.08.01–89312).

## Data analysis

The data obtained in the study were analyzed using the IBM SPSS V23.0. The fitness for normal distribution was examined using the Shapiro–Wilk test. The results of the analysis were presented as descriptive statistics (mean ± SD) and frequency (percentage) distributions. The reliability of the scales and subscales was examined using the Cronbach’s alpha internal consistency coefficient. The level of significance was set at 0.05 in all statistical analyses performed within the scope of the study. The research model was assessed through path analysis using AMOS V24.0. The predictive role of nurses’ perceptions of spiritual care on their attitudes toward caregiving for patients approaching death and dying was examined with path analysis. Path analysis, a type of structural equation model, was used to reveal relationships between the observed variables. The structural equation model did not restrict the number of dependent variables, allowing the examination of the relationship between more than one dependent and one independent variable under certain conditions with a single model. Model fit was evaluated using Chi-Square Index (CMIN/DF), Goodness-Of-Fit Index (GFI), Adjusted Goodness-Of-Fit Index (AGFI), Incremental Fit Index (IFI), the Tucker-Lewis Index (TLI), Comparative Fit Index (CFI), Root Mean Square Error of Approximation (RMSEA), and Standardized Root Mean Squared Residual (SRMR).

## Results

A total of 383 nurses participated in the study. Of the nurses (*n* = 366), the majority were female (67.6%), married (63.4%), had a bachelor’s degree (64.5%), were permanently employed (58.0%), liked their profession (78.9%), and worked in shifts (75.5%). A plurality had been employed for 1–5 years (35.0%), was working in an internal unit (42.8%), and provided care to 6–10 patients per day (33.7%). The mean age of the nurses was 32.9 ± 7.6 (Table 1).

**Table 1.** Distribution of sociodemographic and professional characteristics of the nurses (*n*: 383)

Characteristics	*n*	%
**Age** (Mean ± SD) (32.9 ± 7.6)		
**Gender**		
Female	259	67.6
Male	124	32.4
**Marital status**		
Married	243	63.4
Single	140	36.6
**Education status**		
Vocational High School of Health	51	13.3
Associate degree	60	15.7
Bachelor’s degree	247	64.5
Postgraduate degree	25	6.5
**Duration of employment as a nurse**		
1−5 years	134	35.0
6−10 years	90	23.5
11−15 years	54	14.1
16–20 years	57	14.9
21 years and more	48	12.5
**Unit of employment**		
Intensive care	58	15.1
Surgical unit	121	31.6
Internal unit	164	42.8
Other unit	40	10.4
**Employment type**		
Permanent	222	58.0
Contract	161	42.0
**Status of liking the profession**		
Like	302	78.9
Dislike	25	6.5
Unsure	56	14.6
**Manner of work**		
Continuous daytime	94	24.5
Shift	289	75.5
**Mean number of patients care provided to per day**		
1−5 patients	108	28.2
6−10 patients	129	33.7
11−15 patients	88	23.0
16−20 patients	44	11.5
21−25 patients	8	2.1
26 patients and more	6	1.6

SD: Standard Deviation.

The mean scores of the nurses were 3.4 ± 0.5 for the SSCRS, 47.1 ± 10.5 for the SSPS, 100.2 ± 9.6 for the FATCOD, 4.3 ± 1.1 for the DAP-R *Fear of Death* subscale, 4.1 ± 1.2 for the *Death Avoidance* subscale, 5.5 ± 0.9 for the *Neutral Acceptance* subscale, 4.9 ± 1.0 for the *Approach Acceptance* subscale, and 3.9 ± 1.5 for the *Escape Acceptance* subscale (Table 2).

**Table 2.** Total and subscale scores for the SSCRS, SSPS, FATCOD, and DAP-R

Used scales	X ± SD	Median (Min.–Max)
SSCRS score	3.4 ± 0.5	3.4 (1.5−5.0)
SSPS score	47.1 ± 10.5	47.0 (0−60.0)
FATCOD score	100.2 ± 9.6	99.0 (67.0−137.0)
DAP-R Fear of Death subscale score	4.3 ± 1.1	4.4 (1.3−7.0)
DAP-R Death Avoidance subscale score	4.1 ± 1.2	4.2 (1.3−7.0)
DAP-R Neutral Acceptance subscale score	5.5 ± 0.9	5.8 (1.8−7.0)
DAP-R Approach Acceptance subscale score	4.9 ± 1.0	4.9 (1.0−6.9)
DAP-R Escape Acceptance subscale score	3.9 ± 1.5	4.0 (1.0−7.0)

X: Arithmetic mean; SD.: Standard Deviation; Min.: Minimum; Max.: Maximum; SSCRS: Spirituality and Spiritual Care Rating Scale; SSPS: Spiritual Support Perception Scale; FATCOD: Frommelt Attitude Toward Care of the Dying Scale; DAP-R = Death Attitude Profile-Revised.

The path coefficient between the SSCRS and the DAP-R subscales of *Fear of Death* (β = 0.232; *P* = 0.025), *Death Avoidance* (β = 0.301; *P* = 0.007), *Neutral Acceptance* (β = 0.22; *P* = 0.01), *Approach Acceptance* (β = 0.444; *P* < 0.001), and *Escape Acceptance* (β = 0.659; *P* < 0.001) was statistically significant. The path coefficient between the FATCOD and the DAP-R subscales of *Fear of Death* (β = − 0.032; *P* < 0.001), *Death Avoidance* (β = −0.038; *P* < 0.001), and *Neutral Acceptance* (β = 0.02; *P* < 0.001) was statistically significant. The path coefficient between the SSPS and the DAP-R subscales of *Neutral Acceptance* (β = 0.252; *P* < 0.001) and *Approach Acceptance* (β = 0.162; *P* = 0.026) was statistically significant (Table 3).

**Table 3.** Results of path coefficient analysis showing the relationship between total SSCRS, SSPS, and FATCOD scores and the DAP-R Fear of Death, Death Avoidance, Neutral Acceptance, Approach Acceptance, and Escape Acceptance Subscale Scores

Dependent variable		Independent variable	β^1^	β^2^	S. error	Test stat.	*P*	*R*^2^
DAP-R Fear of Death	< –	SSCRS	0.109	0.232	0.104	2.240	**0.025**	0.091
DAP-R Fear of Death	< –	SSPS	−0.041	−0.066	0.078	−0.839	0.401	
DAP-R Fear of Death	< –	FATCOD	−0.279	−0.032	0.006	−5.720	**<0.001**	
DAP-R Death Avoidance	< –	SSPS	−0.032	−0.056	0.084	−0.669	0.503	0.110
DAP-R Death Avoidance	< –	FATCOD	−0.304	−0.038	0.006	−6.302	**<0.001**	
DAP-R Death Avoidance	< –	SSCRS	0.130	0.301	0.111	2.698	**0.007**	
DAP-R Neutral Acceptance	< –	SSCRS	0.126	0.220	0.085	2.593	**0.010**	0.097
DAP-R Neutral Acceptance	< –	SSPS	0.192	0.252	0.064	3.953	**<0.001**	
DAP-R Neutral Acceptance	< –	FATCOD	0.211	0.020	0.005	4.335	**<0.001**	
DAP-R Approach Acceptance	< –	SSPS	0.110	0.162	0.073	2.224	**0.026**	0.068
DAP-R Approach Acceptance	< –	FATCOD	0.071	0.008	0.005	1.431	0.153	
DAP-R Approach Acceptance	< –	SSCRS	0.227	0.444	0.097	4.591	**<0.001**	
DAP-R Escape Acceptance	< –	SSPS	− 0.031	−0.065	0.104	−0.623	0.533	
DAP-R Escape Acceptance	< –	FATCOD	−0.011	−0.002	0.008	− 0.223	0.824	0.057
DAP-R Escape Acceptance	< –	SSCRS	0.237	0.659	0.138	4.768	**<0.001**	

β^1^: Standardized beta coefficient; β^2^: Non-standardized beta coefficient; S. error.: Standard error; Test stat.: Test statistics; SSCRS: Spirituality and Spiritual Care Rating Scale; SSPS: Spiritual Support Perception Scale; FATCOD: Frommelt Attitude Toward Care of the Dying Scale; DAP-R = Death Attitude Profile-Revised.

**Figure 1.**
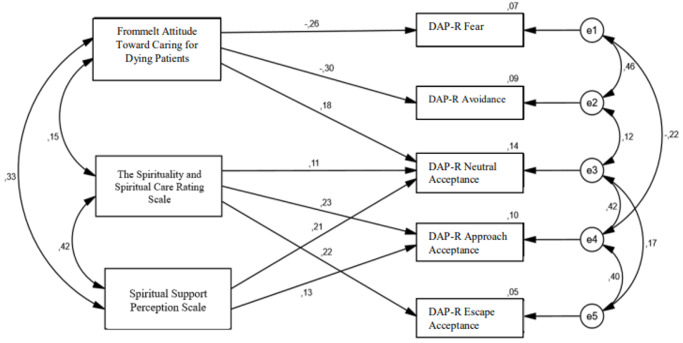
Path diagram showing statistically significant paths. (All values are standardized regression coefficients). DAP-R = Death Attitude Profile-Revised.

The study model was examined with a path analysis. In the model created within the scope of this study, the SSCRS, FATCOD, SSPS mean scores were included as independent variables, while the attitude towards death, which was examined in 5 sub-dimensions – the DAP-R Fear of Death, Death Avoidance, Neutral Acceptance, Approach Acceptance and Escape Acceptance – was included as the dependent variable (Fig. 1). Model fit indices were CMIN/DF (22.735/11) = 2.067, GFI = 0.986, AGFI = 0.953, IFI = 0.979, TLI = 0.945, CFI = 0.979, RMSEA = 0.053, and SRMR = 0.045.

## Discussion

In this study, the path analysis revealed the relationship between the variables of SSCRS, SSPS, FATCOD, and DAP-R, showing that nurses’ high levels of perception of spirituality, spiritual support, and spiritual care, as well as their feelings of being competent to care for individuals approaching death, were important predictors of their attitudes towards death. In addition, this study confirmed Watson’s theory of human care (Watson 2010; Masters 2015).

Watson emphasizes the significance of spiritual care in the relationship between a nurse and a patient. She reports that nurses must provide comfort to patients by acknowledging patients’ own religion and/or sense of spirituality. Her theory emphasizes that the task of nurses is to create meaning in both life and death (Watson 2010; Masters 2015). According to Watson, nurses who practice spiritual care must also ensure that they are providing holistic care that meets the needs of patients. Holistic health care is thus seen to be at the center of nursing care practice and requires nurses to be compassionate towards all patients (Watson 2010; Masters 2015). Nurses need to provide spiritual care that meets the needs of all patients and their families throughout the disease process and in the final stages of the individual’s life (Kavalalı Erdoğan and Koç 2023; Kurtgöz and Koç 2023).

Nurses’ perceptions of spirituality and spiritual care can affect their ability and readiness to provide this kind of care to patients (Melhem et al. 2016). Nurses who want to provide holistic nursing care need to plan the physical care of their patients while also considering these patients’ spiritual care needs. Therefore, it has been recommended that nurses develop their skills in practicing spiritual care by using Watson’s theory of care (Kurtgöz and Koç 2023).

Spiritual care is important not only for palliative care, hospice, and intensive care patients who are usually approaching death, but also in all areas where nursing care is provided (Giske and Cone 2015; Bone et al. 2018). In end-of-life care, nurses can help promote a peaceful death by applying spiritual care (Kavalalı Erdoğan and Koç 2023; Kurtgöz and Koç 2023). Compared to other healthcare professionals, it is easier for nurses to identify individuals’ spiritual care needs because they spend more time with patients (Danacı et al. 2022; Yavuz and Koç 2023). Therefore, nurses need to be aware of their own perceptions of spirituality, so that they can assess their patients’ preferred spiritual resources and coping strategies and provide the appropriate care (Danacı et al. 2022).

Nurses’ perceptions of spiritual care and attitudes toward death have differed in different studies. It has been reported that inadequacies in nurses’ awareness and delivery of spiritual care may be due to deficiencies in nursing education (Bone et al. 2018; Batstone et al. 2020; Rahman et al. 2021), intense workload after graduation, and heavy workloads (Rahman et al. 2021). Studies have reported that the delivery of spiritual care by nurses is associated with spiritual care competence (Li et al. 2021), that nurses’ inadequacies regarding spiritual sensitivity (Byoung Sook and Su Young 2017) and evaluation of the spiritual dimension lead to spiritual care being neglected (Byoung Sook and Su Young 2017; Batstone et al. 2020), and that nurses use interventions that increase the psychological, spiritual, and social comfort of dying patients less than physical interventions (Kisvetrova et al. 2018). Although nurses are responsible for the holistic assessment of their patients and the delivery of holistic care, spiritual care is often not sufficiently integrated into nursing care (Kudubes et al. 2021).

Nurses’ attitudes toward death may affect the delivery of spiritual care. In a study conducted by Kudubes et al. (2021), it was reported that an increase in nurses’ negative attitudes toward death reduced their levels of awareness of spirituality and spiritual care. In another study on this topic, it was determined that half of the nurses had negative attitudes towards the care of dying patients and felt inadequate with regard to the psychological and spiritual dimensions of providing care (Hamdan et al. 2023). In the study of Wang et al. (2018), it was found that nurses’ personal attitudes toward death or negative views about death were associated with their attitudes towards the care of dying patients, that the majority of nurses tended to avoid death and perceived death as natural, and that they considered death as a happy passage to the afterlife.

Providing care to dying patients is challenging and the majority of nurses are not prepared for patient death in the clinic (Becker et al. 2017). Lack of education and experience regarding death or dying patients, attitudes towards death, religious beliefs, and professional limitations may affect nurses’ attitudes towards the care of dying patients (Çevik and Kav 2013; Wang et al. 2018). An important role that nurses play is to provide both physical and spiritual care to dying patients and to support their families (Gurdogan et al. 2017). Therefore, nurses should be aware of the impact of their negative attitudes towards death on the care they provide (Pehlivan et al. 2020).

Attending to a patient’s spiritual needs means caring about them, acknowledging their beliefs and experiences, and helping them with the questions of meaning and of finding hope. There is evidence that spirituality plays a role in coping strategies and positive health behaviors (Astle and Duggleby 2019). Nurses should make use of their knowledge about the individual’s spiritual well-being and engage in interventions that will help maximize the individual’s sense of inner peace and healing (Potter et al. 2017).

### Limitations of the study

This study has some limitations. Since it had a cross-sectional design, causal implications cannot be deduced from the results. The study was conducted in a single hospital. The data obtained were based on the subjective self-reports of the nurses. The hospital where the study was conducted is located in a city. Therefore, it is not known whether the findings would differ in nurses working in rural areas. Furthermore, no data were collected regarding cultural characteristics of nurses that may have affected their perceptions of the concept of spiritual care and attitudes towards death. It is recommended that comparative studies be conducted on this subject with sample groups with different cultural characteristics from different regions.

## Conclusion

In this study, the path coefficient between the SSCRS, SSPS, and FATCOD and the DAP-R subscales of *Fear of Death, Death Avoidance, Neutral Acceptance, Approach Acceptance*, and *Escape Acceptance* was found to be statistically significant. In this regard, it was determined that there was a correlation between nurses’ perceptions of the concept of spiritual care and their attitudes towards death and the care of dying and near-death patients.

## Clinical implications

Nurses need to know the importance of providing spiritual care and be ready to offer death-oriented care to their dying patients in their last moments (Gorchs-Font et al. 2021; Haroen et al. 2023). Nurses should be aware that spirituality is often central to an individual’s sense of self and involves feelings and thoughts that bring purpose and meaning to their life (White et al. 2011). Nurses’ perceptions, awareness and competencies in spiritual care should be increased to meet patients’ spiritual needs and improve their quality of life (Batstone et al. 2020; Li et al. 2021; Wisesrith et al. 2021). Therefore, nurses should be trained in spirituality, spiritual support, and spiritual care. Increasing nurses’ awareness of spiritual support and spiritual care will help them communicate more closely with patients approaching death, more easily identify patients at risk of spiritual distress, and more readily meet patients’ spiritual needs. Nevertheless, nurses should allow patients to share their feelings with them without hesitation. Nurses should make patients approaching death feel valued and cared for. Nurses should take the time to listen to patients in order to better identify their spiritual and spiritual care needs. Nurses should pay attention to the patient’s beliefs and rituals. Nurses must first be aware of their own spirituality in order to integrate spirituality into the care of a patient approaching death (Wilkinson et al. 2016). The meaning that nurses attribute to illness and death should also be explored (Pehlivan et al. 2020). Providing individualized care that is in harmony with the spirituality of healthy and unwell individuals can result in a positive health care experience for both the patient and the nurse (White et al. 2011).

To improve nurses’ awareness of spiritual care and their attitudes towards death and care of dying patients, these topics should be included more prominently in the curricula of nursing schools. In this direction, the implementation of a palliative and end-of-life care curriculum that enables nursing students to understand that death is a natural part of life has been recommended (Österlind et al. 2016; Haroen et al. 2023; Şahan and Kaçmaz 2024). Such a topic should take into account the cultural, spiritual, and religious characteristics of individuals (Jiang et al. 2019; Garcı´a-navarro et al. 2021). Emphasizing the importance of spiritual care during vocational education should help ensure that post-graduate nurses are able to consider the spiritual dimension of each patient and meet their different needs (Jiang et al. 2019). The competencies required by post-graduate nurses when providing spiritual care should be identified, and, if necessary, training programs should be given to improve their awareness of, and attitudes towards, spiritual care (Adip-Hajbaghery et al. 2017).
